# A facile approach to preparing personalized cancer vaccines using iron-based metal organic framework

**DOI:** 10.3389/fimmu.2023.1328379

**Published:** 2024-01-08

**Authors:** Xia Li, Shinya Hattori, Mitsuhiro Ebara, Naoto Shirahata, Nobutaka Hanagata

**Affiliations:** ^1^ Research Center for Macromolecules and Biomaterials, National Institute for Materials Science (NIMS), Tsukuba, Ibaraki, Japan; ^2^ Bioanalysis Unit, Research Network and Facility Services Division, National Institute for Materials Science (NIMS), Tsukuba, Ibaraki, Japan; ^3^ Research Center for Materials Nanoarchitectonics (MANA), National Institute for Materials Science (NIMS), Tsukuba, Ibaraki, Japan; ^4^ Graduate School of Chemical Sciences and Engineering, Hokkaido University, Sapporo, Japan

**Keywords:** cancer immunotherapy, personalized cancer vaccines, metal organic framework, ferric ions, endogenous fumarate ligands, nanotechnology, adjuvants

## Abstract

**Background:**

Considering the diversity of tumors, it is of great significance to develop a simple, effective, and low-cost method to prepare personalized cancer vaccines.

**Methods:**

In this study, a facile one-pot synthetic route was developed to prepare cancer vaccines using model antigen or autologous tumor antigens based on the coordination interaction between Fe^3+^ ions and endogenous fumarate ligands.

**Results:**

Herein, Fe-based metal organic framework can effectively encapsulate tumor antigens with high loading efficiency more than 80%, and act as both delivery system and adjuvants for tumor antigens. By adjusting the synthesis parameters, the obtained cancer vaccines are easily tailored from microscale rod-like morphology with lengths of about 0.8 μm (OVA-ML) to nanoscale morphology with sizes of about 50~80 nm (OVA-MS). When cocultured with antigen-presenting cells, nanoscale cancer vaccines more effectively enhance antigen uptake and Th1 cytokine secretion than microscale ones. Nanoscale cancer vaccines (OVA-MS, dLLC-MS) more effectively enhance lymph node targeting and cross-presentation of tumor antigens, mount antitumor immunity, and inhibit the growth of established tumor in tumor-bearing mice, compared with microscale cancer vaccines (OVA-ML, dLLC-ML) and free tumor antigens.

**Conclusions:**

Our work paves the ways for a facile, rapid, and low-cost preparation approach for personalized cancer vaccines.

## 1 Introduction

In the past decade, cancer immunotherapy has been in the spotlight as an innovative technology that alters the direction of cancer treatment (1–5). Among them, immune checkpoint inhibitors have been approved as first-line drugs in the treatment of diverse cancers (4). However, the response rate for immune checkpoint inhibitor monotherapy remains at 10-40%, because the prerequisite for its effectiveness is the preexistence of large amounts of tumor antigen-specific T cells (4, 6). Therefore, therapeutic cancer vaccines that induce tumor antigen-specific T-cell immune response are expected to be the next breakthrough in cancer immunotherapy (1–3, 7).

Therapeutic cancer vaccines aim to train the patients’ own immune system to recognize and eradicate cancer cells in the body (1–3). Tumor antigens are antigenic substances produced by tumor cells, which are tumor markers to identify and recognize tumor cells. According to the source of tumor antigens, cancer vaccines can be divided into two categories: shared tumor antigens vaccines and personalized tumor antigens vaccines (8–10). Cancer vaccines made from shared antigens (e.g., free peptides) had been the mainstream of clinical research since the 1990s, but their clinical response rates were low, due to tumor heterogeneity, insufficient immunogenicity, susceptibility to tumor antigen loss, and the lack of effective adjuvants (8, 11). Compared with shared tumor antigens vaccines, personalized tumor antigen vaccines generally exhibited much higher response rates (8–10). Personalized tumor antigens vaccines are further classified into predefined personalized antigens vaccines and unidentified personalized antigens vaccines (1–3). Predefined personalized antigens vaccines (e.g., neoantigen vaccines) are associated with extremely high cost, extremely time-consuming preparation process and immune escape of heterogeneous tumors (1–3). While, unidentified personalized antigens vaccines derived from lysed tumor cells may greatly lower the cost of personalized cancer vaccines, and are expected to be less susceptible to tumor antigen loss because they carry large numbers of diverse tumor antigens (8–10).

On the other hand, adjuvants play an important role in enhancing the immunogenicity of tumor antigens and the therapeutic efficacy of cancer vaccines (12–19). Especially, codelivery of antigens and adjuvants in cancer vaccines are expected to trigger a robust antitumor immunity and develop highly efficient cancer vaccines (12, 14, 20, 21). Despite tremendous efforts in the past decades, it remains difficult to prepare personalized cancer vaccines using a facile approach with universal adjuvants and high loading efficiency.

Fumaric acid is an endogenous molecule in the human body, since it is an intermediate in the citric acid cycle, which cells use to generate energy from food in the form of adenosine triphosphate (ATP), and is also a product of the urea cycle (22). Fumarate is widely used in food additives, such as acid regulator, and pharmaceuticals. For example, ferrous fumarate is clinically used to treat iron deficiency anemia (23). Dimethyl fumarate is a drug clinically used to treat the autoimmune diseases psoriasis and multiple sclerosis (24). Tenofovir disoproxil fumarate is an antiviral drug approved by the United States Food and Drug Administration (FDA) for the treatment of chronic hepatitis B virus infection (HBV) and human immunodeficiency virus infection (HIV) (25). Recently, dimethyl fumarate is reported to be highly cytotoxic in cancer cells with KRAS mutation, one of the most common molecular alterations in adult carcinomas (26). In 2023, fumarate is reported to induce the release of mitochondrial DNA into the cytosol, stimulate interferon production, and drive innate immunity (27), which may be associated with immune infiltration in cold tumors. On the other hand, iron elements help strengthen the immune system (28, 29).

In this study, a facile, rapid, and low-cost one-pot route was developed to prepare personalized cancer vaccines by embedding model antigen or autologous tumor antigens within Fe-based metal organic framework through the coordination interaction between Fe^3+^ ions and endogenous fumarate ligands. Herein, Fe-based metal organic framework can effectively encapsulate tumor antigens with high loading efficiency >80%, and act as both delivery system and adjuvants for tumor antigens. By adjusting the synthesis parameters, the morphology of the obtained cancer vaccines is tailored from microscale to nanoscale. Personalized cancer vaccines effectively enhance antigen uptake and Th1 cytokine secretion, strengthen lymph node targeting and cross-presentation of tumor antigens, mount antitumor immunity, and inhibit the growth of established tumor in tumor-bearing mice.

## 2 Materials and methods

### 2.1 Materials

Iron (III) chloride hexahydrate (FeCl_3_·6H_2_O), fumaric acid (C_4_H_4_O_4_) and sodium hydroxide (NaOH) were purchased from Fujifilm Wako, Japan. Ovalbumin (OVA) was purchased from Sigma-Aldrich.

### 2.2 Synthesis of metal organic framework and cancer vaccine

In a typical synthesis, metal organic framework was prepared by mixing fumaric acid (100 mM, 300 μL), NaOH solution (1 M, 30 μL), FeCl_3_·6H_2_O solution (100 mM, 300~600 μL), and water to make the total volume 3 mL with sonication for 30 min, 2 hours, or 4 hours in ice. The resulting products were centrifuged, washed with ultrapure water, dispersed in water for later use or freeze-dried.

Lewis lung carcinoma cells (LLC, Bioresource Research Center, Japan) at 6.7×10^6^ cells/mL were repeatedly frozen and thawed 4 times in minus 30 degrees refrigerator and ice to prepare tumor cell lysate, and then centrifuged at 1500 rpm for 3 min to obtain the supernatant, which was named autologous tumor antigen dLLC.

In a typical synthesis of cancer vaccines, model antigen OVA (50 mg/mL, 12 uL) or autologous tumor antigen dLLC (36 uL) was encapsulated into metal organic framework by mixing fumaric acid (100 mM, 300 μL), NaOH solution (1 M, 30 μL), FeCl_3_·6H_2_O solution (100 mM, 300~600 μL), and water to make the total volume 3 mL with sonication for 30 min or 2 hours in ice.

### 2.3 Quantitative approach of biomolecules loading amounts and samples mass

The concentrations of model antigen OVA or LLC tumor cell lysate in solutions before and after loading are analyzed using a Micro BCA protein assay kit (Thermo Scientific Inc.). The encapsulation efficiencies of proteins are calculated by the following formula, respectively: Proteins encapsulation efficiency = (Initial concentration - Final concentration after encapsulation)/Initial concentration×100%. The mass of metal organic framework or cancer vaccines is calculated by measuring weight of tubes before and after synthesis reaction.

### 2.4 Physicochemical characterization

Morphological observation was carried out using field emission high resolution scanning electron microscope (FE-SEM, Hitachi SU8000, Japan) after being coated with platinum or carbon. The analysis of phases was conducted by a powder X-ray diffractometer with CuKα X-ray (RINT-Ultima III, Rigaku, Japan). The samples were analyzed using Fourier transform infrared spectroscopy (IRTracer-100, Shimadzu, Japan). The zeta potentials were determined using a zeta potential analyzer (ELSZ-1000Z, Otsuka Electronics, Japan). The hydrodynamic diameters were measured using a dynamic light scattering spectrophotometer (DLS-8000HAL, Otsuka Electronics, Japan).

### 2.5 Cellular uptake of OVA antigen and DCs activation *in vitro*

Bone marrow derived DCs were harvested as follows (7). Firstly, bone marrow cells were collected from femurs and tibias of mice (C57BL/6J, CLEA Inc.). After red blood cell lysis and depletion of I-A/I-E-, CD4- and CD8-expressing cells, the residual cells were cultured in RPMI 1640 medium supplemented with 10% fetal bovine serum (FBS) and 20 ng/mL granulocyte macrophage colony-stimulating factor (GM-CSF). The nonadherent and loosely adherent cells were collected as bone marrow derived DCs on day 9-10.

DCs were seeded at a density of 5×10^4^ cell/cm^2^ in a glass bottom dish for several hours. Then, cancer vaccines synthesized using fluorescein conjugated- ovalbumin (F-OVA, Life technologies) were added into the above DCs medium at a final concentration of 30 μg/mL for particle and 5 μg/mL for F-OVA. F-OVA in free format with an equivalent dose was used as control. After overnight culture, cells were stained with lysosome marker (LysoTracker Red) and nuclear staining dye (Hoechst), added with ProLong Live Antifade Reagent to prevent the loss of fluorescent signal due to photobleaching, and observed using a confocal laser scanning microscope (CLSM, Leica TCS SP5).

To further analyze their activation, DCs were seeded onto a flat-bottom 96-well cell culture plate at 2×10^5^ cells/well and then exposed to cancer vaccines suspensions with a particle concentration of 30 μg/mL and an OVA concentration of 5 μg/mL, respectively. OVA in free format was used as control. One day later, the supernatant was collected to quantify the cytokines concentration using enzyme-linked immunosorbent assay kit (ELISA, BD Biosciences).

### 2.6 Lymph node targeting and antigen cross-presentation *in vivo*

Female C57BL/6J mice (CLEA Inc.) were immunized by subcutaneously injecting Fe- based cancer vaccines into the left flank (Fe-based metal organic framework particles, 600 μg/mouse; F-OVA, 100 μg/mouse). An equivalent dose of F-OVA in free format was used as control. Immunized mice were euthanized one day later, and nearby draining lymph nodes were harvested. To analyze the lymph node targeting, the obtained lymph nodes were freshly frozen in Tissue-Tek O.C.T. compound to prepare the cryostat sections. Then, the sections were mounted using SlowFade™ Diamond mountant with DAPI and observed using Leica CLSM. To carry out the analysis about antigen cross-presentation, the obtained lymph nodes were ground through a 70 μm cell strainer to obtain single cell suspension. The obtained cells were blocked with purified anti-CD16/CD32 antibody, and then stained with anti-CD11c-APC and anti-H-2K^b^- SIINFEKL-PE (BioLegend) antibodies. Flow cytometry was performed using a Spectral Cell Analyzer (SP6800, Sony). FlowJo software was used for the analysis of flow cytometry data.

### 2.7 Anti-tumor experiments using E.G7-OVA lymphoma *in vivo*

Twenty female C57BL/6J mice (5~6 weeks old, CLEA Inc.) were randomly divided into four groups. First, E.G7-OVA lymphoma cells (American Type Culture Collection, ATCC; 1.2×10^5^ cells/mouse) were subcutaneously inoculated into the left flanks of mice on day 0. On days 4, 7, and 10 post tumor inoculation, the following substances in 100μL saline were subcutaneously injected into the right flanks of mice according to the divided groups: 1) saline; 2) OVA (100 μg/mouse OVA in free format); 3) OVA-ML (Large-size metal organic framework encapsulated with OVA: 100 μg/mouse OVA and 600 μg/mouse particles); 4) OVA-MS (Small-size metal organic framework encapsulated with OVA: 100 μg/mouse OVA and 600 μg/mouse particles). Tumor size was measured by a caliper and tumor volume was calculated according to the formula: 1/2 × length × width^2^.

At the endpoint, spleen was harvested and triturated through a 70 μm cell strainer to obtain single cell suspension. The cells were stained with anti-CD4-FITC (Biolegend), anti-CD8a-APC/Cyanine7 (Biolegend) and T-Select H-2K^b^ OVA Tetramer-SIINFEKL-APC (MBL) antibodies after the Fc block using purified anti-CD16/CD32 antibody. Flow cytometry was performed using a Spectral Cell Analyzer (SP6800, Sony) and data analysis was carried out with FlowJo software. In addition, the spleen was digested with a tissue protein extraction reagent (Thermo Scientific Inc.) at the same ratio of the tissue weight to extraction reagent, and cytokines contents were determined by ELISA kits (BD Biosciences).

### 2.8 Anti-tumor experiments using Lewis lung carcinoma *in vivo*

Female C57BL/6J mice (5~6 weeks old, CLEA Inc.) were subcutaneously inoculated with Lewis lung carcinoma cells (8×10^4^ cells/mouse) into their left flanks on day 0. On days 4, 7, and 10 post tumor inoculation, the following substances in 100μL saline were subcutaneously injected into the right flanks of mice according to the divided groups: 1) saline; 2) dLLC (6 μL autologous tumor antigens/mouse in free format); 3) dLLC-ML (Large-size metal organic framework encapsulated with autologous tumor antigens: 6 μL/mouse autologous tumor antigens and 600 μg/mouse particles); 4) dLLC-MS (Small-size metal organic framework encapsulated with autologous tumor antigens: 6 μL/mouse autologous tumor antigens and 600 μg/mouse particles). Tumor size was measured by a caliper and tumor volume was calculated according to the formula: 1/2 × length × width^2^.

At the endpoint, spleen was harvested and triturated through a 70 μm cell strainer to obtain single cell suspension. The cells were stained with anti-CD3-APC, anti-CD4-PE/Cyanine7, anti-CD8a-APC/Cyanine7, anti-CD44-FITC and anti-CD62L-PE antibodies (Biolegend) after the Fc block using purified anti-CD16/CD32 antibody. Flow cytometry was performed using a Spectral Cell Analyzer (SP6800, Sony) and data analysis was carried out with FlowJo software.

### 2.9 Statistical analysis

All data are presented as the mean ± standard deviation (SD). Data were analyzed using one-way analysis of variance (ANOVA) followed by Tukey’s multiple comparisons *post hoc* test. A p value less than or equal to 0.05 is considered statistically significant.

### 2.10 Ethical issue

All the animal experiments included in this study have been approved by the Animal Ethics Committee of National Institute for Materials Science (NIMS), Japan. All the animal experimental procedures and animal care were performed in accordance with the guidelines of the Animal Ethics Committee of NIMS, Japan.

## 3 Results

### 3.1 One-pot synthesis of FeMOF-based cancer vaccines

**Figure 1:**
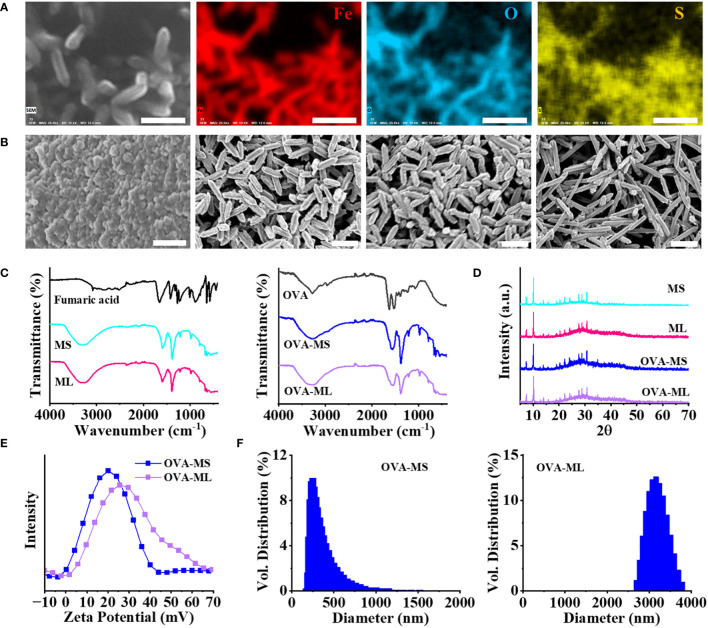
Physicochemical characterization of FeMOF and FeMOF-based cancer vaccines with tailored size. **(A)** EDX mapping analysis of FeMOF-based cancer vaccine (scale bar 1μm). Uniform distribution of Fe, O and S elements evidenced that model antigen OVA was homogeneously embedded in the FeMOF partilces. **(B)** FeMOF-based cancer vaccines with OVA model antigens synthesized in the ratio of Fe^3+^ and fumaric acid at 1:1 for 30 min (OVA-MS, Left 1, scale bar 500nm), 2 hours (OVA-ML, Left 2, scale bar 1μm), and 4 hours (Left 3, scale bar 1μm). FeMOF-based cancer vaccines with OVA model antigens synthesized in the ratio of Fe^3+^ and fumaric acid at 2:1 for 4 hours (Left 4, scale bar 1μm). **(C)** FTIR spectra of fumaric acid and FeMOF with different sizes (MS, ML) (Left). FTIR spectra of OVA model antigens and FeMOF -based cancer vaccines with OVA model antigens (OVA-MS, OVA-ML) (Right). **(D)** XRD patterns of FeMOF and FeMOF-based cancer vaccines. **(E)** Zeta potentials of FeMOF -based cancer vaccines. **(F)** Particle size distribution of samples FeMOF -based cancer vaccines.

Fe-based metal organic framework (FeMOF) was synthesized by mixing fumaric acid, NaOH solution, FeCl_3_·6H_2_O solution, and water with sonication in ice. To prepare personalized cancer vaccines, OVA model antigens or autologous LLC lysates were supplemented and encapsulated into FeMOF during the synthesis process. Energy dispersive X-ray (EDX) mapping analysis of cancer vaccines suggest that S elements -containing OVA model antigens are homogeneously embedded in the formed FeMOF particles (**Figure 1A**).

The obtained cancer vaccines ranged from microscale to nanoscale by adjusting the synthesis parameter, such as concentration of raw materials (**Figure 1B**). FeMOF-based cancer vaccines with OVA model antigens, synthesized in ice with the final ratios of FeCl_3_·6H_2_O and fumaric acid at 1:1 for 30 min and 2 hours, exhibit nanoparticle-like morphology with sizes of about 50~80 nm and rod-like morphology with lengths of about 0.8 μm, respectively, which are named OVA-MS and OVA-ML, respectively. When the reaction time is further extended to 4 hours, FeMOF-based cancer vaccines show the same rod-like morphology of about 0.8 μm as that of 2 hours. When the final ratios of FeCl_3_·6H_2_O and fumaric acid were changed from 1:1 to 2:1, FeMOF-based cancer vaccines synthesized in ice for 4 hours exhibit rod-like morphology with lengths of about 1.5 μm.

Fourier transform infrared spectroscopy (FTIR) confirms the formation of Fe-fumarate coordination compounds and the encapsulation of OVA model antigens within them (**Figure 1C**). Fumaric acid shows strong C=O stretching band near 1655 cm^-1^, which is attributed to its carboxylic acid group. FeMOF samples without the presence of antigens, including MS and ML, exhibit the absorption bands near 1585 cm^-1^ and 1382 cm^-1^, which are attributed to asymmetric and symmetric stretching modes in the carboxyl group of metal fumarates (**Figure 1C**, Left). FeMOF-based cancer vaccines with OVA model antigens, including OVA-MS and OVA-ML, show similar absorption bands near 1585 cm^-1^ and 1382 cm^-1^ with FeMOF, suggesting the formation of metal fumarates (**Figure 1C**, Right). In addition, the shoulder absorption bands near 1635 cm^-1^ in OVA-MS and OVA-ML suggest the encapsulation of OVA model antigens in FeMOF-based cancer vaccines, since OVA model antigens show strong C=O stretching bands near 1635 cm^-1^ (**Figure 1C**, Right).

Wide-angle X-ray powder diffraction (XRD) patterns of FeMOF and FeMOF-based cancer vaccines with different sizes indicate the formation of Fe-based metal organic framework MIL-88A (**Figure 1D**). The zeta potentials of FeMOF-based cancer vaccines OVA-MS and OVA-ML are centered at 21 and 27 mV, respectively (**Figure 1E**). Dynamic light scattering (DLS) measurements suggest that the hydrodynamic sizes of OVA-MS and OVA-ML are approximately 300 nm and 3 μm, respectively (**Figure 1F**), which is larger than the particle size directly observed by SEM due to the partial aggregation of particles in the solution without dispersant.

The concentrations of model antigen OVA or LLC tumor cell lysate in solutions before and after loading into FeMOF were analyzed using a Micro BCA protein assay kit. The encapsulation efficiencies of OVA in FeMOF-based cancer vaccines OVA-MS and OVA-ML are about 90% and 91%, respectively. While the encapsulation efficiencies of autologous LLC lysates in FeMOF-based cancer vaccines dLLC-MS and dLLC -ML are about 80% and 82%, respectively.

### 3.2 Size-dependent antigen uptake by DCs and their activation *in vitro*

**Figure 2:**
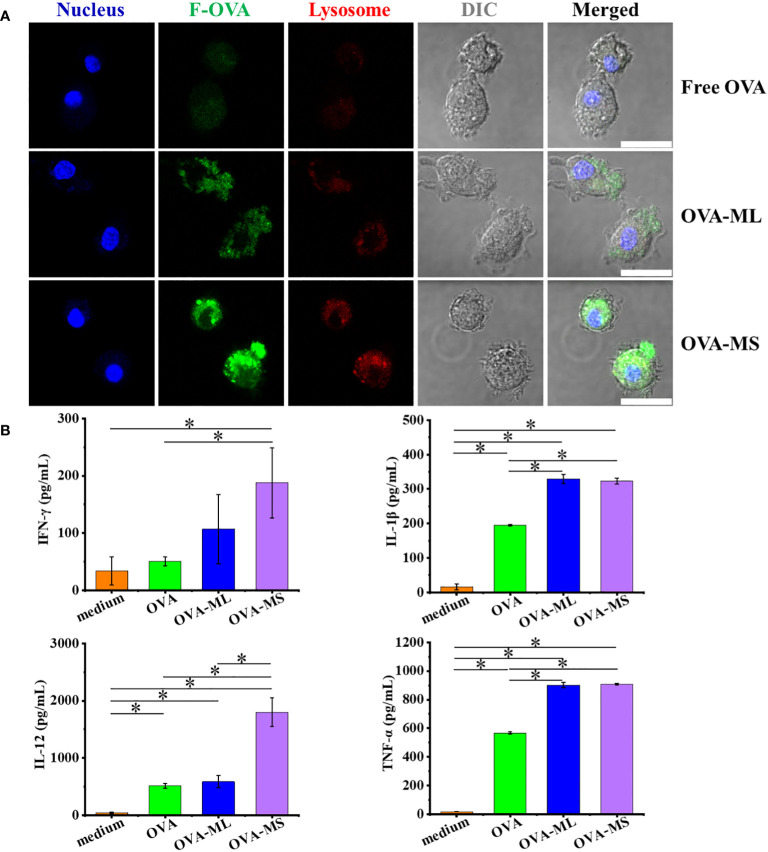
FeMOF-based cancer vaccines effectively enhance antigen uptake and activation of DCs *in vitro*. **(A)** Representative confocal laser scanning microscope images of dendritic cells after culture with free F-OVA and vaccines overnight with lysosome staining (scale bar 20μm). **(B)** Quantitative analysis of DCs activation after culture with free OVA and self-assembled vaccines for 1 days. Data in b, n=3 independent samples, one-way ANOVA followed by Tukey’s multiple comparisons *post hoc* test, p<0.05. All data are presented as mean ± SD.

DCs are the most professional antigen-presenting cells to uptake tumors antigens and trigger an adaptive immune response. In this study, primary bone marrow derived DCs from mice were cocultured with as-prepared cancer vaccines overnight to test the cellular uptake of antigens and antigen-presenting cells activation. Herein, fluorescein conjugated - ovalbumin (F-OVA) with green fluorescence was used to prepare cancer vaccines with visualized antigens. F-OVA solutions in free format was used as control group. Small-size cancer vaccines (OVA-MS) present much higher green fluorescence intensity than large-size cancer vaccines (OVA-ML) and free F-OVA control group, as shown in CLSM images (**Figure 2A**). The images suggest that F-OVA molecules embedded in small-size particles can be more effectively captured by DCs than those embedded in large-size particles and those in free format.

Further, when DCs were cocultured with cancer vaccines embedded with OVA model tumor antigens for one day, their activation was assessed using ELISA assay (**Figure 2B**). DCs cocultured with FeMOF-based cancer vaccines show significantly higher cytokines secretion, such as interleukin (IL) -1β and tumor necrosis factor (TNF) -α, compared with free OVA and medium groups. DCs cocultured with small-size cancer vaccines more efficiently promote the cytokines secretion, including IL-12 and interferon (IFN) -γ, compared with large-size cancer vaccines, free OVA, and medium groups.

### 3.3 FeMOF-based cancer vaccines in small size significantly strengthen lymph node targeting and cross-presentation of tumor antigens *in vivo*

**Figure 3:**
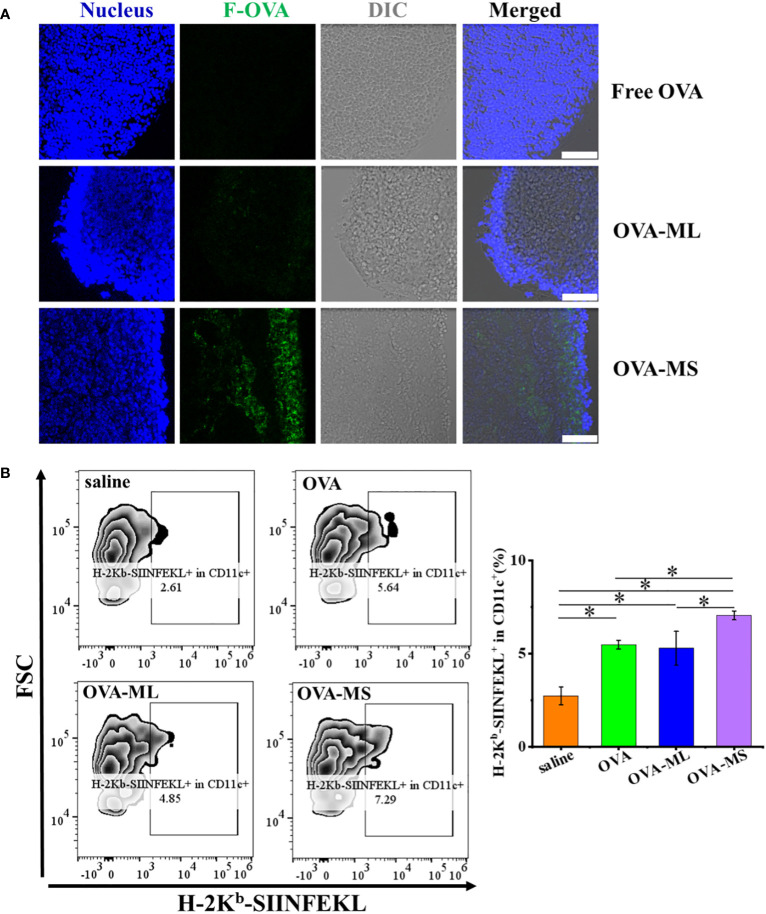
FeMOF-cancer vaccines in small size significantly enhance lymph node targeting and cross-presentation of tumor antigens *in vivo*. **(A)** Representative confocal laser scanning microscope images of lymph nodes in mice administrated with free F-OVA and FeMOF-based cancer vaccines with large size and small size (OVA-ML and OVA-MS, scale bar 50μm). **(B)** Representative flow cytometry plots of H-2K^b^-SIINFEKL^+^ in CD11c^+^ cells population in lymph nodes of mice vaccinated with free OVA and FeMOF-based cancer vaccines (Left). Quantitative analysis of H-2K^b^-SIINFEKL^+^ in CD11c^+^ cells population in lymph nodes of mice vaccinated with free OVA and FeMOF-based cancer vaccines (Right). Data in b, right, n=3 independent animals, one-way ANOVA followed by Tukey’s multiple comparisons *post hoc* test, p<0.05. All data are presented as mean ± SD.

To test the lymph node targeting abilities of tumor antigens in different vaccine formulations *in vivo*, the obtained vaccines synthesized using fluorescein conjugated- OVA model antigens with green fluorescence were subcutaneously injected into C57BL/6J mice, and the draining lymph nodes were collected 16 hours later. Then the cryosections of the draining lymph nodes were prepared and observed by CLSM. As shown in **Figure 3A**, small-size cancer vaccines groups (OVA-MS) exhibit higher green fluorescence intensity of fluorescein conjugated- OVA model antigens than free model antigen group and large-size cancer vaccines (OVA-ML). The results suggest that small-size cancer vaccines more significantly enhance the lymph node targeting of tumor antigens *in vivo*, compared with large-size cancer vaccines and those in free format.

Then, cross-presentation of OVA model tumor antigens *in vivo* were quantitatively investigated by flow cytometry using H-2K^b^-SIINFEKL^+^ in CD11c^+^ cell populations in lymph node as evaluation indicators (**Figure 3B**). CD11c is a commonly used as cell marker for mouse DCs. Herein, H-2K^b^-SIINFEKL^+^ in CD11c^+^ cell populations in lymph node represent cross-presentation of OVA tumor antigens by DC cells. FeMOF-based cancer vaccines in small size (OVA-MS) significantly enhance cross-presentation of tumor antigens *in vivo*, compared with all the other groups, including saline group, free OVA group and FeMOF-based cancer vaccines in large size (OVA-ML).

The lymph node targeting of tumor antigens and their subsequent antigen cross-presentation play a very critical role in inducing tumor antigen-specific immunity. In this study, FeMOF-based cancer vaccines in small size is the most effective among all groups in enhancing the lymph node targeting of tumor antigens and strengthening their cross-presentation *in vivo*.

### 3.4 Antitumor effects of FeMOF-based vaccines in therapeutic mouse E.G7-OVA lymphoma model

**Figure 4:**
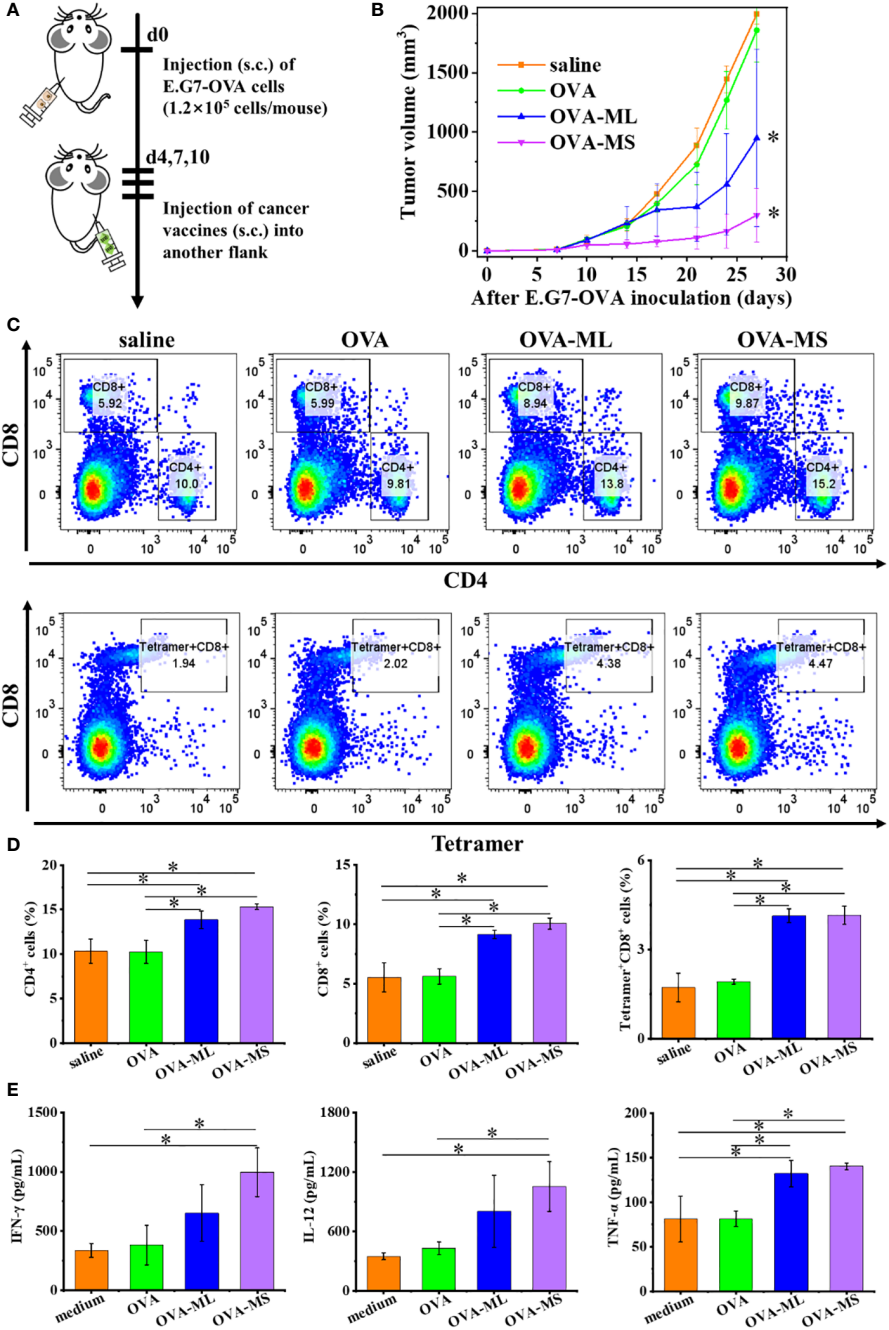
Antitumor effects of FeMOF-based cancer vaccines in therapeutic mouse E.G7-OVA lymphoma model. **(A)** Schematic illustration of antitumor experiments: E.G7-OVA lymphoma cells (1.2×10^5^ cells/mouse) were inoculated subcutaneously into the left flank of female C57BL/6J mice; On days 4, 7, and 10 post tumor inoculation, FeMOF-based cancer vaccines were injected into the right flank of mice; Tumor growth was continuously measured. **(B)** Average tumor growth curves of different vaccines formulations. Data in b, n=5 independent animals, one-way ANOVA followed by Tukey’s multiple comparisons *post hoc* test, p<0.05. All data are presented as mean ± SD. **(C-D)** Representative flow cytometry plots **(C)** and populations **(D)** of CD4^+^, CD8^+^ and tetramer^+^CD8^+^ T cells in spleen at the endpoint. **(E)** Quantitative analysis of cytokines in spleen at the endpoint. Data in d and e, n=3 independent animals, one-way ANOVA followed by Tukey’s multiple comparisons *post hoc* test, p<0.05. All data are presented as mean ± SD.

Twenty female C57BL/6J mice were randomly divided into four groups. E.G7-OVA lymphoma cells (1.2×10^5^ cells/mouse) were inoculated subcutaneously into the left flank of mice to establish the therapeutic tumor model (**Figure 4A**). On days 4, 7, and 10 post tumor inoculation, FeMOF-based cancer vaccines in large size (OVA-ML) and small size (OVA-MS) in 100μL saline were subcutaneously administrated into the right flank of mice. In addition, only saline and free OVA in saline were administrated as control groups. Later, tumor size was continuously measured to study the therapeutic effect of tumor vaccines on distant tumors (**Figure 4B**). Saline group and free OVA group show considerable and rapid tumor growth of E.G7-OVA lymphoma. However, mice treated with FeMOF-based cancer vaccines exhibited the inhibition in tumor growth, compared with saline and free OVA groups. Especially, FeMOF-based cancer vaccines in small size (OVA-MS) more effectively inhibited the tumor growth of E.G7-OVA lymphoma than those in large size (OVA-ML). The therapeutic effects of FeMOF-based cancer vaccines on E.G7-OVA lymphoma suggest that FeMOF is not only a delivery system for OVA model tumor antigens, but also an effective adjuvant to trigger anti-tumor immune response.

To investigate the underlying antitumor mechanism of FeMOF-based cancer vaccines, the spleens of mice at the endpoint of antitumor experiments were collected to check CD4^+^, CD8^+^ and tetramer^+^CD8^+^ T cell populations (**Figures 4C, D**). The CD4^+^, CD8^+^ and tetramer^+^CD8^+^ T cell populations in splenocytes in FeMOF-based cancer vaccines in large size (OVA-ML) and small size (OVA-MS) are significantly higher than saline group and free OVA group. The average CD4^+^ and CD8^+^ T cell populations in small-size cancer vaccines (OVA-MS) are higher *those in* in large-size cancer vaccines (OVA-ML), although no significant difference is observed. Moreover, the cytokines in spleen were determined by ELISA assay (**Figure 4E**). Small-size cancer vaccines (OVA-MS) more efficiently stimulated the secretion of IFN-γ and IL-12, compared with other groups.

### 3.5 Antitumor effects of FeMOF-based vaccines in therapeutic mouse Lewis lung carcinoma model

**Figure 5:**
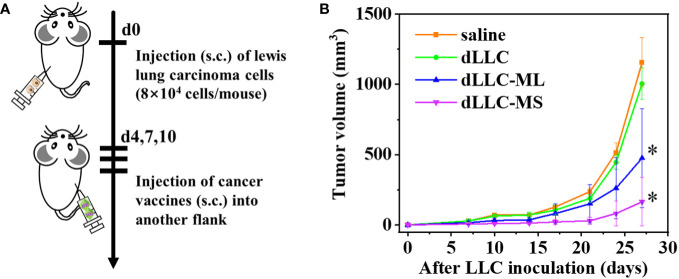
Antitumor effects of FeMOF-based cancer vaccines in therapeutic mouse Lewis lung carcinoma model. **(A)** Schematic illustration of antitumor experiments: Lewis lung carcinoma cells (8×10^4^ cells/mouse) were inoculated subcutaneously into the left flank of female C57BL/6J mice; On days 4, 7, and 10 post tumor inoculation, FeMOF-based cancer vaccines were injected into the right flank of mice; Tumor growth was continuously measured. **(B)** Average tumor growth curves of different vaccines formulations. Data in b, n=4 independent animals, one-way ANOVA followed by Tukey’s multiple comparisons *post hoc* test, p<0.05. All data are presented as mean ± SD.

Female C57BL/6J mice were randomly divided into four groups. LLC cells (8×10^4^ cells/mouse) were inoculated subcutaneously into the left flank of mice to establish the therapeutic tumor model (**Figure 5A**). On days 4, 7, and 10 post tumor inoculation, FeMOF-based cancer vaccines in large size (dLLC-ML) and small size (dLLC-MS) in 100μL saline were subcutaneously administrated into the right flank of mice. Saline group and dLLC autologous tumor antigens in free format were used as controls. Later, tumor sizes were monitored to confirm the therapeutic effect of FeMOF-based personalized cancer vaccines towards lewis lung carcinoma in the distant sites (**Figure 5B**). As shown in the curves, only dLLC autologous tumor antigens in free format did not inhibit the tumor growth of Lewis lung carcinoma, compared with free saline group. While FeMOF-based cancer vaccines encapsulated with dLLC autologous tumor antigens effectively inhibited tumor growth, compared with saline and free dLLC autologous tumor antigens groups. Moreover, FeMOF-based cancer vaccines in small size (dLLC-MS) more effectively inhibited the tumor growth than those in large size (dLLC-ML). In general, FeMOF-based cancer vaccines encapsulated with dLLC autologous tumor antigens show the same tendency to inhibit tumor growth as those encapsulated with OVA model tumor antigens.

**Figure 6:**
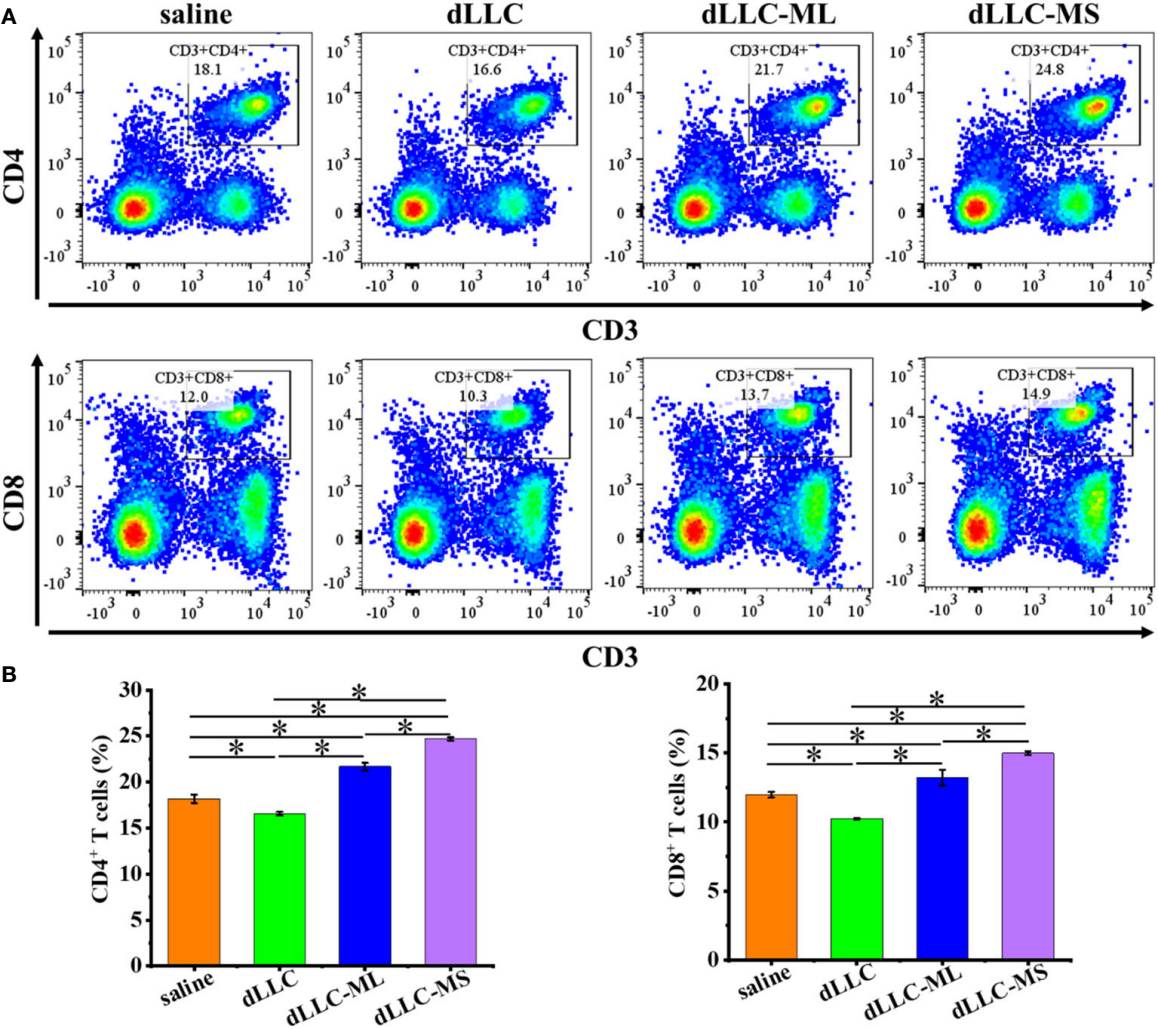
Antitumor mechanism analysis of FeMOF-based cancer vaccines in therapeutic mouse Lewis lung carcinoma model. Representative flow cytometry plots **(A)** and populations **(B)** of CD4^+^ and CD8^+^ T cells in spleen at the endpoint. Data in b, n=4 independent animals, one-way ANOVA followed by Tukey’s multiple comparisons *post hoc* test, p<0.05. All data are presented as mean ± SD.

**Figure 7:**
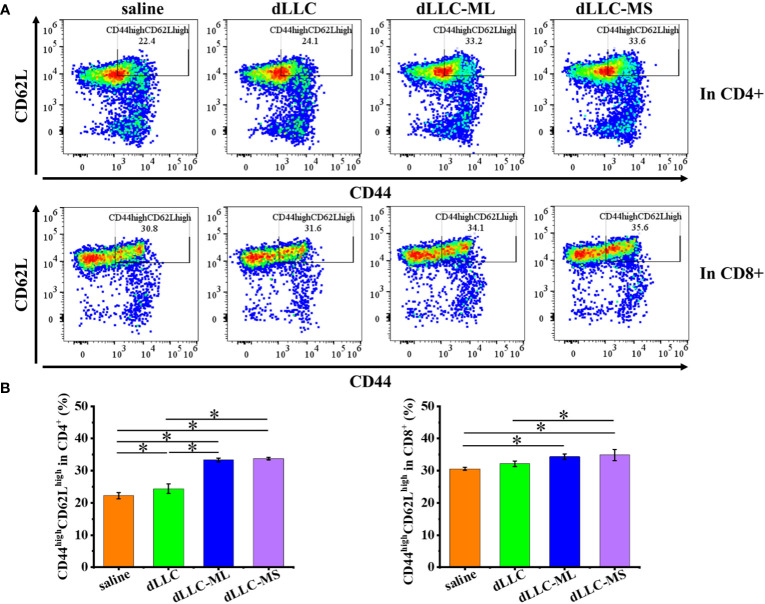
Antitumor mechanism analysis of FeMOF-based cancer vaccines in therapeutic mouse Lewis lung carcinoma model. Representative flow cytometry plots **(A)** and populations **(B)** of CD44^high^CD62L^high^ in CD4^+^ and CD8^+^ T cells in spleen at the endpoint. Data in b, n=4 independent animals, one-way ANOVA followed by Tukey’s multiple comparisons *post hoc* test, p<0.05. All data are presented as mean ± SD.

To investigate the underlying antitumor mechanism of FeMOF-based cancer vaccines encapsulated with dLLC autologous tumor antigens, the spleens of mice at the endpoint of antitumor experiments were collected to analyze CD4^+^, CD8^+^, CD44^high^CD62L^high^ in CD4^+^, and CD44^high^CD62L^high^ in CD8^+^ T cell populations (**Figures 6**, **7**). The CD4^+^ and CD8^+^ T cell populations in splenocytes in FeMOF-based cancer vaccines in large size (dLLC-ML) and small size (dLLC-MS) are significantly higher than saline group and free dLLC autologous tumor antigens group (**Figure 6**). More importantly, the average CD4^+^ and CD8^+^ T cell populations in small-size cancer vaccines (dLLC-MS) are higher *those in* in large-size cancer vaccines (dLLC-ML). To further analyze the immunological memory responses induced by FeMOF-based cancer vaccines, the central memory T cells (CD44^high^CD62L^high^ in CD4^+^ or CD8^+^) in the spleens in various groups were tested by flow cytometry. The percentage of the central memory T cells in mice treated with FeMOF-based cancer vaccines, such as CD44^high^CD62L^high^ in CD4^+^ T cells, is much higher than that those treated with saline or free dLLC autologous tumor antigens (**Figure 7**).

## 4 Discussion

Due to their tunable composition, versatile structure, and diverse functions, metal organic frameworks have attracted increasing attention in biomedical field, such as drug delivery system, cancer therapy, imaging and so on. Metal organic frameworks (MOF) are constructed from the coordinating self-assembly of metal moieties with organic ligands, which can be exogenous or endogenous. In this study, endogenous fumarate ligands were employed together with iron ions, one of the most important minerals in humans, to prepare iron-based metal organic frameworks with good biocompatibility. As we mentioned in the introduction part, fumaric acid is an important intermediate product of the citric cycle in the body, which is a source of intracellular energy in the form of ATP (22). Fumarate is recently reported to stimulate interferon secretion and trigger innate immunity (27), which has the potential to enhance the immune infiltration in cold tumors. On the other hand, iron plays a pivotal role in the innate and adaptive immunity, such as macrophage polarization, natural killer cells activity, T cells activity and so on (30). Iron deficiency leads to the inhibition of T cells proliferation and antibody immune response (30). Herein, FeMOF built from iron ions and endogenous fumarate ligands not only serves as a delivery system for tumor antigens, but also acts as an intrinsic adjuvant to enhance the immune response to tumor antigens. Moreover, in this study, the aqueous green synthesis route in ice with no use of toxic organic solvents is adopted to achieve the one-pot synthesis of FeMOF-based cancer vaccines with high encapsulation efficiency larger than 80% and effectively maintains the immunogenicity of tumor antigens.

FeMOF-based cancer vaccines are promising for both predefined personalized antigens vaccines and unidentified personalized antigens vaccines. In this study, two distinct mouse tumor models, including mouse E.G7-OVA lymphoma model with predefined tumor antigen and Lewis lung carcinoma models with unidentified tumor antigens, were used to evaluate the therapeutic efficacy of FeMOF-based cancer vaccines. Herein, FeMOF efficiently encapsulates these two personalized tumor antigens, such as OVA model tumor antigens and dLLC autologous tumor antigens, with encapsulation efficiency higher than 80%. We demonstrated the concept of one-pot FeMOF-based cancer vaccines using these two completely different types of tumor cells. This approach is expected to be a universal method that can be applied to other types of tumors simply by replacing tumor cell lysates or designated tumor antigens.

*In vitro* assay using primary DCs and *in vivo* analysis of cytokines in spleen suggest that FeMOF materials exhibit intrinsic adjuvant properties by promoting the secretion of Th1 cytokines, such as IL-12, TNF-α, and IFN-γ. IL-12 is a proinflammatory cytokine, which primarily produced by antigen-presenting cells, such as DCs, macrophages, monocytes and so on (31). IL-12 exhibits multiple immunomodulatory functions, such as inducing the differentiation of Th0 into Th1 lymphocytes, increasing the cytolytic activation of NK cells and cytotoxic T lymphocytes, and promoting the secretion of TNF-α and IFN-γ by T cells, all of which facilitates to transform the immunosuppressive cold tumors into immunologically active hot tumors (31). TNF-α is predominantly produced by activated antigen-presenting cells and is a powerful tumoricidal cytokine, as its name describes, which induces the apoptotic cell death, stimulates the inflammatory response at the tumor sites and inhibits the tumorigenesis (31). IFN-γ not only exhibits immunomodulatory functions on innate and adaptive immune response, but also has direct cytotoxic effects on tumor cells (31). Although these kinds of Th1 cytokines, including IL-12, TNF-α, and IFN-γ, are promising in cancer immunotherapy, their clinical application is associated with short half-life, narrow therapeutic window, severe dose-limiting toxicities, and difficulties in large-scale manufacturing (31). In this study, FeMOF adjuvant materials may induce antigen-presenting cells to secrete Th1 cytokines *in situ*, stimulate the secretion of Th1 cytokines in immune organs and trigger systemic activation of antitumor immunity, which provides another way to use cytokines in cancer immunotherapy.

FeMOF-based cancer vaccines based on different tumor antigens effectively trigger T-cells immune response in different kinds of tumor-bearing mice, including E.G7-OVA lymphoma model and Lewis lung carcinoma model. Vaccination using FeMOF-based cancer vaccines efficiently increase the cell populations of typical T lymphocytes in immune organs, such as CD4^+^, CD8^+^ cells and so on. CD4^+^ T cells play a prominent role in adaptive immunity, which are traditionally considered to provide help for CD8^+^ cells to trigger antitumor immune response, and recently reported to also own direct antitumor capacity (32). CD8^+^ T cells, often called cytotoxic T lymphocytes, are the major drivers of antitumor immunity and have the capacity to selectively detect and eliminate cancer cells. In E.G7-OVA lymphoma model, OVA tumor antigens-specific tetramer^+^CD8^+^ T cells have also been analyzed, and the results suggest that FeMOF-based cancer vaccines resulted in the increase in tumor antigens-specific CD8^+^ T cells populations. On the other hand, in Lewis lung carcinoma model, the central memory T cells (CD44^high^CD62L^high^ in CD4^+^ or CD8^+^) have been quantified, which suggests that FeMOF-based cancer vaccines may enhance immune memory.

Due to time and space limitations, the present study focuses on evaluating the anti-tumor efficacy of FeMOF-based cancer vaccines with tailored morphology using two different tumor models, and their effects on immune response in spleen and lymph. The effects of cancer vaccines on the tumor microenvironment are not involved. According to existing literature reports, we have reason to believe that the rationally designed cancer vaccines may have a great impact on the tumor microenvironment, such as the infiltration of tumor-specific T cells, the macrophages polarization towards M1 type, the inhibition of regulatory T cells (Treg) and myeloid-derived suppressor cells (MDSC) and so on (33–35). Further research on the above contents will be conducted in the future.

## 5 Conclusions

In summary, a rapid one-pot synthetic route has been developed to synthesize personalized cancer vaccines using model antigen or autologous tumor antigens based on the coordination interaction between Fe^3+^ ions and endogenous fumarate ligands. Herein, Fe-based metal organic framework can effectively encapsulate tumor antigens with high loading efficiency >80%, and act as both delivery system and adjuvants for tumor antigens. By adjusting the synthesis parameters, the morphology of the obtained cancer vaccines is easily tailored from microscale to nanoscale. When cocultured with antigen-presenting cells, nanoscale cancer vaccines more effectively enhance antigen uptake and Th1 cytokine secretion than microscale ones. Nanoscale cancer vaccines more effectively enhance lymph node targeting and cross-presentation of tumor antigens, mount antitumor immunity, and inhibit the growth of established tumor in tumor-bearing mice, compared with microscale cancer vaccines. Our approach to developing personalized cancer vaccines has the potential to be applied to other tumor types by replacing tumor cell lysates or designated tumor antigens.

## Data availability statement

The raw data supporting the conclusions of this article will be made available by the authors, without undue reservation.

## Ethics statement

The animal study was approved by The Animal Ethics Committee of National Institute for Materials Science (NIMS), Japan. The study was conducted in accordance with the local legislation and institutional requirements.

## Author contributions

XL: Conceptualization, Data curation, Formal analysis, Funding acquisition, Investigation, Methodology, Project administration, Resources, Writing – original draft, Writing – review & editing. SH: Methodology, Writing – review & editing. ME: Methodology, Writing – review & editing. NS: Methodology, Writing – review & editing. NH: Methodology, Supervision, Writing – review & editing.
